# Evaluation of the learning curve for robotic single-anastomosis duodenal–ileal bypass with sleeve gastrectomy

**DOI:** 10.3389/fsurg.2022.969418

**Published:** 2022-07-22

**Authors:** Lun Wang, Yang Yu, Jinfa Wang, Shixing Li, Tao Jiang

**Affiliations:** Department of Bariatric and Metabolic Surgery, China-Japan Union Hospital of Jilin University, Changchun, China

**Keywords:** learning curve, robotic, single-anastomosis duodenal-ileal bypass with sleeve gastrectomy, SADI-S, textbook outcome

## Abstract

**Background:**

The robotic surgical system is being used in various bariatric procedures. However, only a few studies with very small sample size are present on robotic single-anastomosis duodenal–ileal bypass with sleeve gastrectomy (SADI-S). Moreover, to date, the learning curve of robotic SADI-S has been poorly evaluated yet.

**Objective:**

This retrospective study aimed to estimate the learning curve of robotic SADI-S.

**Methods:**

102 consecutive patients who underwent robotic SADI-S between March 2020 and December 2021 were included. Textbook outcome standard was performed to comprehensively evaluate clinical outcome of robotic SADI-S. Based on the textbook outcome, we evaluated the learning curve of robotic SADI-S by the cumulative sum (CUSUM) method.

**Results:**

The mean operative time was 186.13 ± 36.91 min. No conversion to laparotomy or deaths occurred during the study period. The rate of complications was 6.9% (*n* = 7), of which major complications were identified in 2.9% (*n* = 3), including 2 gastric leakages and 1 respiratory failure. A total of 60 patients reached the textbook outcome standard. The rate of textbook outcome was positive and was steadily increasing after the number of surgical cases accumulated to the 58th case. Taking the 58th case as the boundary, all the patients were divided into the learning stage group (the first 58 patients) and mastery stage group (the last 44 patients). The rate of complications, proportion of abdominal drainage tubes and postoperative hospital stay were significantly higher in the learning stage group compared with the mastery stage group (*P *< 0.05). No significant difference was observed between the two groups in terms of patient demographic data, operative times, reoperations and readmission.

**Conclusion:**

Robotic SADI-S is a feasible and reproducible surgical technique with a learning curve of 58 cases.

## Introduction

Compared with conventional laparoscopy, the robotic surgical system offers several advantages (1–8), including 7 degrees of freedom, tremor filtration, three-dimensional high-definition visualization, and superior ergonomics, which might contribute to improve surgical outcomes. Since Cadiere reported the world's first robotic bariatric surgery in 1999, the robotic surgical system has been used in bariatric procedures such as sleeve gastrectomy (SG), Roux-en-Y gastric bypass (RYGB) and biliopancreatic diversion with duodenal switch (BPD/DS) (9–12). In recent years, the robot surgical system begins to be applied in single-anastomosis duodenal–ileal bypass with sleeve gastrectomy (SADI-S), but only a few studies with very small sample size are present (13–16). Moreover, to date, the learning curve of robotic SADI-S has been poorly evaluated yet.

## Materials and Methods

### Patient and clinical data

102 consecutive patients who underwent robotic SADI-S between March 2020 and December 2021 were included in this study. Any revisional operations were excluded. All the surgeries were performed by the same surgeon. We recorded and analyzed the following factors: patient gender, age, preoperative weight, body mass index, waistline, standard live volume, American Society of Anesthesiology Physical Status Classification, operative time, the proportion of abdominal drainage tube, length of postoperative stay, complications, conversion to laparotomy, mortality, reoperation, and readmission. The Dindo–Clavien classification was used to classify the severity of complications, and major complications were defined as grade III or above (17, 18).

### Operative technique

The Da Vinci Xi^®^ model was used for robotic SADI-S. The patient undergoing surgery was in reverse Trendelenburg position with open legs and arms. The first trocar for the 30° camera (8 mm, robotic arm 3) was placed at the lower edge of the navel. Other trocars were inserted under visual inspection. The second trocar for the stapler insertion (12-mm, robotic arm 1) was placed at the junction of the right anterior axillary line and right end of the greater curvature of the stomach. The third trocar for liver retractor (8 mm, robotic arm 2) was placed at the junction of the right midclavicular line and costal margin. The fourth trocar (8-mm, robotic arm 4) was placed at the junction of the left midclavicular line and left end of the greater curvature of the stomach. All the trocars were spaced more than 8 cm, avoiding each other's interference of robotic arms. A 300-cm common channel was measured retrograde from the ileocecal valve and marked by sutures. Sleeve gastrectomy was performed about 4 cm from the pylorus over a 34 Fr bougie tube. Complete duodenum transection was performed about 2 cm from the pylorus. Finally, duodenal–ileal anastomosis was performed continuously by using an absorbable 3-0 barbed suture.

### Moving average method

The moving average method is a simple smoothing forecasting technique. According to time series, item by item, sequential time averages containing a certain number of items are sequentially calculated to reflect the long-term trend (19). We applied the method to the operative time of robotic SADI-S with a moving average order of 20 cases by the following formulate:

MA(n)=(Xn+Xn−1+Xn−2+......+Xn−19)/20

### Definitions

Textbook outcome (TO) was performed to comprehensively evaluate clinical outcome of robotic SADI-S. According to a review of existing “Textbook Outcome” metrics in the literature (20–24), the definition of TO was subsequently expanded for robotic SADI-S-specific outcomes selected based on clinician consensus among a team of bariatric surgeons at our institution. The final definition of TO in robotic SADI-S included the following 5 key parameters: the operative time less than or equal to the 75th percentile of overall operation time (210 min); the postoperative hospital stay less than or equal to the 75th percentile of overall postoperative hospital stay (6 days); complication grade lower than Dindo–Clavien grade II; no conversion to laparotomy, and no rehospitalization or death after robotic SADI-S. TO was recorded when all of the aforementioned parameters were observed.

### Analysis of learning curve for robotic SADI-S

According to the order of operation date, all patients are numbered 1–102. Based on the TO standard, the clinical outcomes of all patients are classified and quantified, that is, the clinical outcome is represented by 1 when it meets the TO standard; otherwise, it is represented by –1. We calculated the rate of TO by the cumulative sum analysis (CUSUM) method. The curve was drawn by case number as x-axis and CUSUM (TO rate) as y-axis so as to understand the learning curve of robotic SADI-S.

### Statistical analysis

SPSS 22.0 was used for statistical analysis. Measurement data were expressed as mean ± standard deviation and were analyzed by the independent-sample Student's t-test (Normality data) or Mann–Whitney U test (skewed data), as appropriate. The calculated data were analyzed by the *χ*^2^ test. A *P*-value of <0.05 was considered statistically significant.

## Results

102 consecutive patients who underwent robotic SADI-S were included in this study and the overall follow-up rate was 100%. Among the 102 patients, 57 were women and 45 were men with a mean age of 34 years (range, 17–61 years). The patient demographic data are summarized in Table 1.

**Table 1:** Patient demographics.

Factor	All patients (*n *= 102)
Gender: Male/Female	45/57
Mean age (years)	34.00 ± 8.61
Preoperative body weight (kg)	122.19 ± 24.98
Preoperative BMI (kg/m^2^)	41.77 ± 6.84
Preoperative waistline (cm)	127.49 ± 15.57
Previous upper abdominal surgery	0
ASA Classification	
Grade II	69 (67.6%)
Grade III	33 (32.4%)

ASA Classification: American Society of Anesthesiology Physical Status classification.

Overall, mortality and conversion to laparotomy did not occur in this study. Seven patients (6.9%) suffered through complications, including two with gastric leakage (2.0%), one with duodenal-ileal anastomotic leakage (1.0%), one with respiratory failure (1.0%), one with postoperative abdominal bleeding (1.0%), one with seroperitoneum (1.0%), and one with delayed gastric emptying (1.0%). Among the seven patients with complication, major complications were identified in 2.9% (*n* = 3), including 2 gastric leakages and 1 respiratory failure. The rates of reoperation and readmission were 2.0% and 2.9%, respectively. Four patients suffered through grade II complications classified on the basis of the Dindo–Clavien classification (one with seroperitoneum, one with postoperative abdominal bleeding, one with delayed gastric emptying, and one with duodenal-ileal anastomotic leakage), and all of them were cured successfully by the conservative treatment. Two patients suffered from gastric leakage (grade IIIb) and required reoperation. One patient was transferred to the intensive care unit because of respiratory failure (grade IV) and was eventually cured.

**Figure 1:**
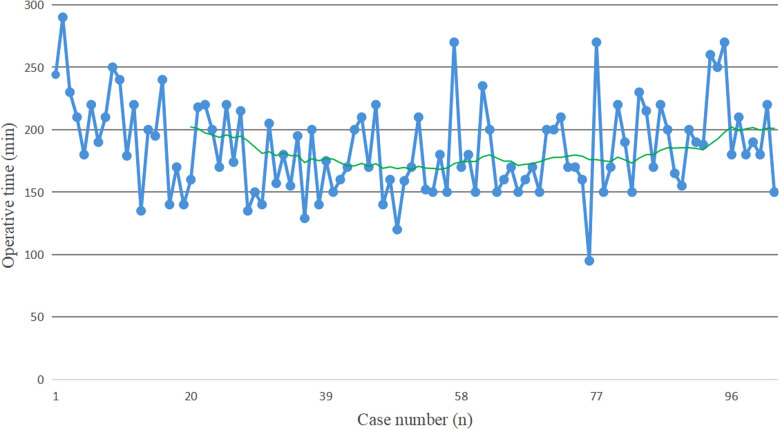
The blue solid points indicate the operative time for each patient in chronological case order. The green line represents the moving average of operative time, which is used to help determine the trend of operative time. In the initial period of developing robotic SADI-S, the slope of operative time goes down most sharply, then tends towards stable.

The raw data of operative time were plotted as blue solid points in chronological case order (Figure 1). Along with the increase of the number of surgical cases, an overall downward trend for operative time was observed. The moving average method showed that in the initial period of developing robotic SADI-S, the slope of operative time went down most sharply, then tended towards stable (The green line in Figure 1).

**Figure 2:**
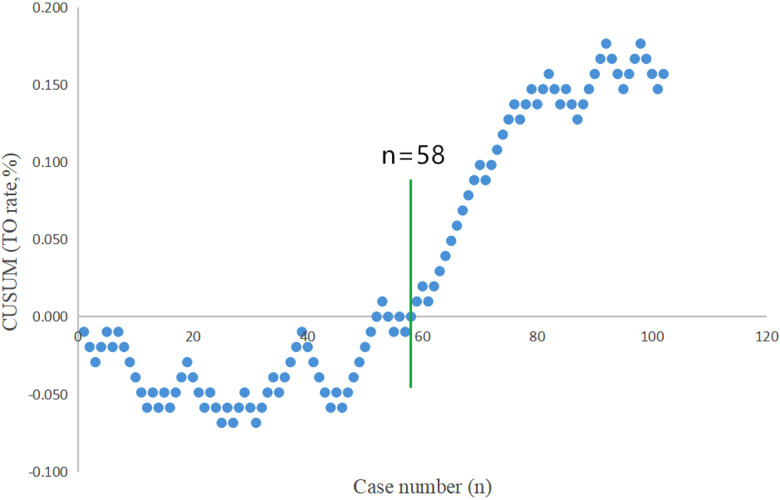
“Textbook outcome (TO)” for robotic SADI-S was defined by all of the following 5 parameters: operative time ≤210 min, the length of postoperative stay ≤6 days, postoperative morbid event <Clavien grade II, no conversion to laparotomy, and no rehospitalization or death after totally robotic SADI-S. TO was recorded when all of the aforementioned parameters were observed. The rate of TO for robotic SADI-S is positive and is steadily increasing after the number of surgical cases accumulated to the 58th case.

The CUSUM plot indicated that the rate of textbook outcome, as previously outlined, was positive and was steadily increasing after the number of surgical cases accumulated to the 58th case (Figure 2). This suggests that the learning curve of totally robotic SADI-S was 58 cases. Subsequently, all the patients were classified into the learning stage group (the first 58 patients) and mastery stage group (the last 44 patients). No significant difference was observed between the learning stage group and mastery stage group in terms of patient's demographic data (Table 2). Except for the rates of complication, proportion of abdominal drainage tubes and lengths of postoperative hospital stay, no significant difference was observed between the learning stage group and mastery stage group in terms of operation-related outcomes (Table 3).

**Table 2:** Comparison of the demographics between the 2 study groups.

	Learning stage group (*n* = 58)	Mastery stage group (*n* = 44)	Statistic	*P*-value
Gender (Male/Female)	25/33	20/24	X^2 ^= 0.056	0.813
Age (years)	34.53 ± 9.24	33.32 ± 7.75	t = 0.705	0.482
Preoperative body weight (kg)	124.98 ± 28.75	118.51 ± 18.59	t = 1.376	0.172
Preoperative BMI (kg/m^2^)	42.69 ± 7.60	40.56 ± 5.55	t = 1.633	0.106
Preoperative waistline (cm)	128.12 ± 17.08	126.65 ± 13.46	t = 0.471	0.638
Standard live volume (cm^3^)	1633.49 ± 211.69	1601.54 ± 141.11	t = 0.913	0.364
ASA Classification (grade II/grade III)	38/20	31/13	X^2 ^= 0.279	0.598

ASA Classification: American Society of Anesthesiology Physical Status classification. BMI: Body mass index.

**Table 3:** Comparison of the operation-related outcomes between the 2 study groups.

	Learning stage group (*n* = 58)	Mastery stage group (*n* = 44)	Statistic	*P*-value
Operative time (min)	185.03 ± 37.59	187.57 ± 36.37	t = −0.342	0.733
Complications overall	7 (12.1%)	0	–	0.018
Conversion to laparotomy	0	0	–	–
Reoperations	2 (3.4%)	0	–	0.505
Readmission^a^	3 (5.2%)	0	–	0.257
Mortality	0	0	–	–
Abdominal drainage tube	26 (44.8%)	5 (11.4%)	X^2 ^= 13.24	0.000
Postoperative hospital stay (days)	7.83 ± 7.07	5.45 ± 1.02	U = 709.00	0.000

^a^ Including two patients with gastric leakage who needed to be reoperated.

## Discussion

SADI-S is being used as a surgical procedure in the treatment of morbid obesity for 14 years since it was first proposed in 2007 by Torres et al. as a simplified procedure of BPD/DS (25). SADI-S is preferred over BPD/DS because of the reduced operative risk by eliminating one anastomosis and similar weight loss and remission of metabolic diseases. Nonetheless, there are still some technical challenges for surgeons to perform SADI-S by laparoscopy because of patient's large waistline, large liver, thick abdominal walls, and substantial visceral fat. Several studies have proved that the advantages of robotic surgical system can contribute to reduce the complication rates and increase the safety of surgery compared with conventional laparoscopy (26–30). Similarly, the robotic surgical system might be helpful in performing SADI-S.

To our knowledge, this study is the first to estimate the learning curve of robotic SADI-S by multifactorial analysis. According to our results, the learning curve of totally robotic SADI-S is 58 cases and can be divided into Phase 1 and Phase 2. Phase 1 includes the first 58 patients and represents the initial learning stage. Phase 2 represents the mastery stage, with a significant reduction in the rate of complications, proportion of abdominal drainage tube and postoperative hospital stay. The learning curve of other bariatric procedures has been reported previously. Previous studies (10, 31) have shown that the learning curve of robotic SG is 20–25 cases. The learning curve for robotic RYGB ranged from 14 to 84 cases (11, 32–35). Sudan et al. (12) reported that the learning curve for robot-assisted BPD/DS was 50 cases.

This study showed that robotic SADI-S is a feasible and safe surgical approach for morbid obesity. A total of 7 patients developed complications (6.9%). In addition to two patients with gastric leakage (grade IIIb) requiring reoperation, the other 5 patients with the complications were cured by the conservative treatment. In general, surgeons are concerned about a higher complication rate during the initial learning phase of the learning curve, wherein they plan to develop a new procedure. In this study, although statistical difference was observed in terms of morbidity between the first 58 patients (learning stage) and the last 44 patients (mastery stage) (12.1% vs. 0%; *P* = 0.018), robotic SADI-S for patients with obesity is relatively safe during the initial phase of the learning curve.

The mean total operative time in this study was 186 min, which is within the range of those reported previously for robotic SADI-S (145–204 min) (13–16). An increase in surgeon's proficiency with the increase in surgical experience was reflected in the operative time required; of note, the slope was more pronounced in the initial phase. However, in the late period of developing robotic SADI-S, we observed an abnormal increase in operative time in some patients, resulting in a significant elevation of the moving average curve. This can be explained by the fact that we measured the length of small intestine twice using two different methods during operation. In this study, the results of Pearson correlation analysis indicated that operative time was negatively correlated with assistant's experience (r = −0.214, *P* = 0.031). However, our study showed that mean operative time didn't decrease in mastery stage group compared with learning stage group. The reason for this difference is associated with the use of new assistants during the mastery stage. Our results showed that postoperative hospital stay was more than five days in both groups, which didn't display the principles of ERAS. The reason for this prolonged admission is that SADI-S is a restrictive and malabsorptive bariatric operation. It is quite possible that patients have trouble taking in adequate liquids after SADI-S, resulting in dehydration. For this, in our center, all patients need to take extra several days for intravenous hydration. Of course, we also timely deal with symptoms caused by food intolerance during this period.

The main limitation of robotic surgery is the perceived higher cost compared with that of laparoscopy. Most previous studies reported that the use of a robotic surgical system increases the cost of the procedure (36–38). However, Hagen et al. (39) reported that the overall cost of robotic RYGB is less compared with laparoscopy. We did not analyze the cost in our study; however, more safety may equalize higher cost. Moreover, the cost of robotic surgery is not necessarily higher than conventional laparoscopy (39).

This study is the first to evaluate the learning curve of robotic SADI-S by multifactorial analysis. However, the study has some limitations. On the one hand, it is a retrospective study. One the other hand, there is no control group in this study. Randomized controlled trials using large sample sizes are required for further study.

## Conclusion

Robotic SADI-S is a feasible and reproducible surgical technique with a learning curve of 58 cases.

## Data Availability

The original contributions presented in the study are included in the article/Suplementary Material, further inquiries can be directed to the corresponding author/s.
